# Molecular Pathogenesis of Alzheimer’s Disease: Reductionist *versus* Expansionist Approaches

**DOI:** 10.3390/ijms10031386

**Published:** 2009-03-26

**Authors:** Rudy J. Castellani, Xiongwei Zhu, Hyoung-Gon Lee, Mark A. Smith, George Perry

**Affiliations:** 1 Division of Neuropathology, University of Maryland, Baltimore, Maryland, USA; 2 Department of Pathology, Case Western Reserve University, Cleveland, Ohio, USA; 3 College of Sciences, University of Texas, San Antonio, Texas, USA

**Keywords:** Alzheimer’s disease, amyloid-β, oxidative stress, tau

## Abstract

Alzheimer’s disease (AD) is characterized clinically by dementia and pathologically by two hallmark lesions, senile plaques and neurofibrillary tangles. About a quarter century ago these hallmark lesions were purified and their protein constituents identified, precipitating an avalanche of molecular studies as well as substantial optimism about successful therapeutic intervention. In 2009, we now have copious knowledge on the biochemical cascades that produce these proteins, the different modifications and forms in which these proteins exist, and the ability to selectively target these proteins for therapeutic intervention on an experimental basis. At the same time, there has been no discernible alteration in the natural course of AD in humans. While it may be that the complexity of AD will exceed our capacity to make significant treatment progress for decades or more, a paradigm shift from the reductionism that defines amyloid-β and tau hypotheses, to one that more accurately reflects the meaning of neuropathological changes, may be warranted. We and others have demonstrated that AD pathology is a manifestation of cellular adaptation, specifically as a defense against oxidative injury. As such, AD pathology is therefore a host response rather than a manifestation of cytotoxic protein injury, and is unlikely to be a fruitful target for therapeutic intervention. An “expansionist” view of the disease, we believe, with oxidative stress as a pleiotropic and upstream process, more aptly describes the relationship between various and numerous molecular alterations and clinical disease.

## 1. Introduction

“Researchers have already cast much darkness on the subject, and if they continue their investigations, we shall soon know nothing at all about it.” - Mark Twain

Alzheimer’s disease (AD) is a heterogeneous condition, differing in age at onset, clinical symptomatology, extent and distribution of pathological changes, presence or absence of germline mutations, presence or absence of polymorphic susceptibility alleles, and presence or absence of a long list of risk factors. This being the case, it is predictable that numerous hypotheses with “treatment implications” have been suggested, and perhaps equally so that none of these hypotheses have resulted in a tangible treatment benefit. The failure of the medical science community to impact the natural course of disease may reflect the complexity of AD, although at some point it becomes reasonable to question the prevailing paradigms and suggest a fundamental re-organization of the thought processes to more directly address cause, and avoid copious and detailed characterizations of epiphenomena [1].

At present, dominant hypotheses equate the soluble protein constituents of insoluble pathological lesions with toxicity. For example, AD brains accumulate neurofibrillary tangles, neurofibrillary tangles contain phosphorylated tau, phosphorylated tau exists in a soluble intermediate form that is toxic to synapses, phosphorylated tau thus is a product of a pathological cascade that is inherently deleterious, and that pathological cascade, if left unchecked, causes neurodegeneration [2]. Alternatively, AD brains accumulate senile plaques, senile plaques contain amyloid-β protein (Aβ), Aβ protein exists in soluble intermediate form that is toxic to synapses, Aβ is therefore a product of a pathological cascade that is inherently deleterious, etc. [3]. Whereas these approaches have a reductionist appeal and are easily understood, they are also presumptuous. Of concern is the poor correlation of these molecules with disease and the increasing literature demonstrating their role in cellular adaptation and survival [4].

In this review, we discuss the molecular pathophysiology of two major proteins implicated in AD pathogenesis, as well as the role of oxidative stress, and the concept of pathology (and associated proteins) as a downstream response in neurodegenerative disease.

## 2. Amyloid-β

Aβ was initially identified through purification of structural pathology-amyloid plaque cores and blood vessels involved by cerebral amyloid angiopathy [5,6]. Identification of Aβ as a metabolic product of amyloid-β protein precursor (AβPP), its position on chromosome 21, and the identification of familial AD kindreds carrying pathogenic AβPP mutations solidified Aβ as a putative central molecule in AD pathogenesis.

While the Aβ cascade concept has shifted somewhat in recent years to reflect soluble Aβ species and targeting of the synapse, it is worth remembering that the current, extensive knowledge of Aβ was derived from the targeting of insoluble pathological lesions, lesions that were, in turn, targeted because they were presumed toxic [1]. The poor correlation between insoluble lesions and disease has rendered the microscopic pathology partly decorative and non-toxic epiphenomena, so the central importance currently of soluble species, being the product of non-toxic insoluble epiphenomena, is remarkably fortuitous.

Aβ is derived from the much larger AβPP, an integral type I membrane glycoprotein encoded in 19 exons on chromosome 21q21 (reviewed in [7–9]. When fully expressed, AβPP contains a short cytoplasmic tail, a transmembrane portion, and a relative large extracellular domain, although in culture only about 10% of newly synthesized AβPP molecules reach the cell surface. AβPP exists as multiple alternatively spliced isoforms, with three predominating: two isoforms, AβPP770/751 contain the so-called Kunitz protease inhibitor domain (exon 7) within the extracellular portion and are considered the predominant forms in non-neuronal cells, whereas AβPP695 is devoid of the Kunitz protease inhibitor domain and is the predominant neuronal form.

Enzyme cleavage is said to result in amyloidogenic and non-amyloidogenic fragments, depending on whether or not processing results in the Aβ protein. Inherent in the term “amyloidogenic” is a process that is fundamentally pathological, although it is worth noting that Aβ synthesis, including the so-called pathogenic Aβ, Aβ_1–42_, is a physiological process, being synthesized and secreted throughout life. In fact, Aβ is detected in healthy controls, as well as AD patients, in cerebrospinal fluid and plasma [10].

Cleavage of AβPP by either α-secretase or β-secretase produces soluble N-terminal fragments AβPP, and C83 and C99 membrane-bound C-terminal fragments, respectively. Further cleavage by γ-secretase leads to the release and secretion of non-pathogenic p3 peptide (previous α-secretase cleavage) and Aβ (previous β-secretase cleavage). Depending on the precise site of γ-secretase cleavage, different lengths of Aβ are produced, varying from 38 to 43 amino acids. The 42 amino acid form, Aβ_42_, has a greater tendency to form fibrils *in vitro* compared to other forms, and therefore comprises the core of the amyloid cascade hypothesis.

The combination of β-secretase and γ-secretase cleavage, resulting in the Aβ peptide, was established early on in the study of Aβ metabolism, while the elucidation of the constituents and cell biology of γ-secretase has proven a challenge, owing to substantial complexity and the interplay with presenilins, PS1 and PS2. The presenilis, initially identified through linkage to early onset familial AD with apparent increased Aβ_42_ [11–13], are two candidates for the components of the γ-secretase complex [14].

Despite the presumption of presenilin as an important component of the multimeric secretase complex, the biochemical mechanism of presenilin action is unknown. During development, presenilins appear to cleave a transmembrane protein called Notch, which in turn is a transcriptional activator of genes involved in cellular differentiation [15]. PS1 and PS2 have been found to be involved in a range of biological processes, including cell adhesion, G-protein mediated signal transduction, the unfolded protein response [16–19]. Nicastrin has also been shown to interact strongly with the presenilins and appears to be required for normal Notch signaling in *Caenorhabditis elegans* [20].

AβPP cleavage with generation of Aβ fragments is also complicated by AβPP processing as a function of cellular subcompartment in which it occurs. Once at the cell surface, AβPP is proteolytically processed, primarily by α-secretases, resulting in shedding of the majority of the extracellular domain within minutes of reaching the plasma membrane. Rapid and efficient internalization is mediated by a “YENPTY” internalization motif near the carboxy terminus of the AβPP molecule [7]. Interestingly, YENPTY mutations decrease Aβ generation. Once endocytosed, AβPP may be recycled to the cell surface, degraded, or further processed. β-site AβPP cleaving enzyme-1 (BACE1) appears to act on AβPP in late Golgi/TGN and endosomes, as supported by the acidic optimal pH of BACE1. γ-secretase complex activity on the other hand apparently takes place in multiple cellular compartments including endoplasmic reticulum, Golgi, and the plasma membrane, although the latter is thought to comprise only a small fraction of the γ-secretase activity.

The cellular function of AβPP is incompletely resolved. One candidate ligand, secreted neuronal protein F-spondin believed to function in neuronal sprouting, binds AβPP as well as APLP-1 and APLP-2, possibly interfering with β-secretase cleavage and cytoplasmic domain mediated cell signaling [8]. AβPP may also serve as a receptor for intracellular transport of synaptic vesicles through interaction with kinesin and microtubules [21]. Both AβPP and the low density lipoprotein receptor-related protein bind the adaptor protein Fe65 via their cytoplasmic domains which increases AβP proteolytic processing, suggesting a relationship between the two proteins [22]. Both LDL receptor-related protein and AβPP are also γ-secretase substrates once their extracellular domains are removed. Perhaps the most important role of AβPP and Aβ is that of an antioxidant in an organ system that is particularly vulnerable to oxidative stress (see below).

In short according to the amyloid cascade hypothesis, the fundamentally toxic Aβ_42_, otherwise a product of normal cellular metabolism, is overproduced in disease and causes neurodegeneration. Support for this concept comes principally from Mendelian diseases with pathogenic AβPP mutations leading to extensive Aβ deposits and early onset disease, presenilin mutation cases that increase the Aβ_42/40_ ratio, and *in vitro* toxicity of Aβ_42_ peptides. Whether Aβ_42_ is toxic in vivo remains to be elucidated, whereas significant evidence for the role of Aβ_42_ in neuroprotection has been demonstrated [4].

### 2.1. Aβ and clinical disease

The relationship between Aβ pathology and disease has been the subject of a number of studies and is well known to be imprecise at best [1]. An early study in the 1960s that showed an overall trend toward increased disease severity with plaque burden [23] has been refuted in a number of subsequent studies [24–26]. At present, it is generally accepted that amyloid burden overall correlates poorly with disease severity, whereas the distribution of Aβ tends to be diffuse throughout the neocortex with little region specificity [25]. Diffuse deposits of amyloid also occur in the striatum and cerebellar cortex [27,28], in the absence of overt loss of function subserved by those regions, and in the absence of neurofibrillary degeneration, either in the form of dystrophic neurites or neurofibrillary tangles. Relative to neocortical Aβ, the medial temporal allocortical region which among other things subserves episodic declarative memory, shows *decreased* Aβ [25], a finding that is often overlooked in studies of synaptic function in the hippocampus. Given the putative role of apolipoprotein E (ApoE) in facilitating fibrillogenesis, it is also interesting that the extent of neocortical Aβ deposits does not correlate with the various ApoE alleles, including ɛ4 [29].

The lack of eloquence of Aβ deposition with respect to clinical signs has led to the suggestion that the more region specific tau pathology is a form of retrograde degeneration, i.e. that the close correlation with neurofibrillary degeneration in the temporal allo- and periallocortex, and memory loss, may be due to Aβ deposits elsewhere, to which phospho-tau-altered neurons are projecting [30]. The opposite sequence has also been suggested with regard to degeneration of the basal nucleus of Meynert and the locus ceruleus—that these regions degenerate early and project to neocortical areas that subsequently develop amyloid deposits [31]. These interesting concepts are as yet unproven, as is the possibility of a temporal and spatial disconnect between Aβ and tau deposits [32], but taken together, studies of regional distribution of proteinaceous deposits highlight the problematic nature of reductionist theories in conditions as complex as AD.

### 2.2. Aβ attack on the synapse

Synapse loss has long been a well known, yet not widely studied, structural condition tightly associated with the cognitive decline in AD. Early work documented a categorical relationship with dementia [33], a relationship which more recently was found to parallel cases of mild AD [34]. In addition, cases assessed to have mild cognitive impairment (MCI) show an intermediate level of synapse loss, again reflecting MCI as preempting the development of AD. In these studies, however, synapse loss showed no relation to senile plaque density or to Braak stage. A relatively new paradigm that is more functional than structural has emerged, rendering moot, presumably, the anatomically imprecise and poorly correlated Aβ deposits. It is now suggested that soluble, low-n Aβ oligomers, identified by high speed centrifugation and Western blotting of the supernatant, cause synaptic damage and functional neurologic deficits [3]. Experimental studies involving injection of conditioned medium, derived from oligomer-secreting AβPP V717F Chinese hamster ovary cells, into rat lateral ventricle, demonstrated alterations in long term potentiation (LTP) that was related to low-n oligomers *per se* and not monomers [35]. LTP alteration was also shown *in vitro* in hippocampal mouse slices, along with concomitant changes in cell cycle signaling cascades and behavioral abnormalities. These models provide useful platforms for analyzing the Aβ-mediated synapse loss, which may be indicative of dysfunction of NMDA-type glutamate receptor activation. Therapeutics blocking this activation directly affect the synaptic profile, as does preventing the formation of amyloid oligomers [36]. These studies support the Aβ immunization strategies that, so far, have been ineffective. On the other hand, the occurrence of an untoward T-cell driven meningoencephalitis in an initial clinical trial is well known [37].

## 3. Phosphorylated Tau

### 3.1. The neurofibrillary tangle

While plaques were a known accompaniment of senile dementia in the late 1800s, the first description of the neurofibrillary tangle in 1907 can be attributed to Alzheimer [38]. It is also interesting to note that Alzheimer devoted ten sentences and two paragraphs to his initial description of the neurofibrillary tangle, compared to only two sentences to the senile plaque [38,39]; this, and the fact that plaques were a known component of senile dementia at that time [40], suggests that the neurofibrillary tangle was the more intriguing lesion. Nevertheless, in spite of copious literature written by Alzheimer and his contemporaries, it is difficult to find firm allusions to the cause of the basic disease process. Rather, the importance and controversy rested for the most part in whether this condition affecting a relatively young patient represented a new disease or, instead, was a form of senile dementia with early onset [41].

Not long after the purification of amyloid deposits and the identification of the Aβ protein, neurofibrillary tangles were purified and the microtubule associated protein tau determined as the major protein component [42]. The enthusiasm for tau phosphorylation as a primary process in AD was however blunted by the absence of genetic linkage, which has forever relegated pathological tau events to a downstream position on the popular algorithms. Instead, germline tau mutations are more closely related to the frontotemporal dementia phenotype [43].

### 3.2. Tau protein

Since the identification of tau as the major component of neurofibrillary pathology, knowledge of tau has expanded considerably. We now know that the tau gene is comprised of over 100 kb and contains 16 exons (reviewed in [44]). Upstream of the first exon are consensus binding sites for transcription factors such as AP2 and SP1. Alternative splicing of tau nuclear RNA transcribed in the adult brain on exons two, three, and ten, results in six tau isoforms. The isoforms differ in the presence of either three of four peptide repeats of 31 or 32 residues in the C termimal region encoded on exon 10. This peptide repeat region comprises the microtubule binding domain. Tau isoforms also differ in the expression of zero, one, or two inserts encoded on exons two and three. The relative amounts of these tau isoforms as well as their phosphorylation status changes during development; 3 repeat tau with no inserts is expressed in the fetus and early postnatal infant, while heterogeneous isoforms are expressed in the adult brain. This switch in RNA splicing also corresponds to a reduction in tau phosphorylation. Tau is relatively abundant in neurons but is present in all nucleated cells and functions physiologically to bind microtubules and stabilize microtubule assembly for polymerization.

In disease, tau is abnormally hyperphosphorylated at proline directed serine/threonine phosphorylation sites [44–46], including Ser-202/Thr-205 (AT8 site), Ser-214 and/or Ser-212 (AT100 site), Thr-231 and/or Ser-235 (TG3 site), and Ser-396/Ser-404 (PHF-1 site). In addition, alternative tau splicing as noted above has a tendency to differ depending on pathological phenotype, such that tau accumulation in AD is a mixture of 3R and 4R tau, Pick disease tends to be 3R tau, corticobasal degeneration and progressive supranuclear palsy tends to be 4R tau, and so-called argyrophilic grain disease accumulates small inclusions comprised of 3R tau. The general term “tauopathy” is thus used as a means of broad classification of neurodegenerative diseases.

### 3.3. Tau and clinical disease

In spite of the fact that tau pathophysiology is generally considered a secondary or downstream phenomenon, it is nevertheless interesting that neurofibrillary pathology correlates closely with clinical signs [26]. The eloquence of phosphorylated tau deposition, for example its proclivity for the memory circuitry in the medial temporal lobe perirhinal, entorhinal, and hippocampal regions early in the disease and in the setting of MCI, indicates a much closer functional relationship to disease than Aβ, at least in terms of spatial distribution of insoluble deposits [47,48]. It is also remarkable that neocortical neurofibrillary pathology is virtually always associated with clinical signs of AD [48], whereas extensive neocortical Aβ deposits are often seen in the absence of significant cognitive impairment or evidence of neuronal loss in the elderly population [24]. Therefore, whereas the pathology of normal aging and that of AD differ more quantitatively than qualitatively, heavy tau “burden” is generally incompatible with preserved cerebral function, while heavy amyloid burden is often tolerated.

### 3.4. Tau attack on the synapse

Not to be outdone, the role of phosphorylated tau in functional disease is progressing in a manner very much similar to Aβ. Once considered toxic due to its insolubility, recent studies indicate that insoluble tau accumulation is somewhat benign and instead implicate soluble, oligomeric phospho-tau intermediates, and indeed their effect on the synapse, as an underlying theme. Experimental support includes a transgenic model in which mice expressing a repressible human tau show behavioral improvements with tau suppression yet continue to accumulate neurofibrillary tangles, indicating that neurofibrillary tangles themselves are insufficient to cause cognitive decline or neuronal death [49]. In another AD-like model, axonal pathology with accumulation of tau preceded plaque deposition [50]. Studies of a P301S tauopathy model demonstrated microglial activation and synapse loss prior to neurofibrillary tangle formation implicating inflammatory response as a possible therapeutic target for maintaining synapse well-being [51]. These studies, along with others inhibiting protein phosphatases which also result in synapse loss as a result of increased tau phosphorylation [52] indicate that neurofibrillary tangle formation is a late stage non-toxic event and directing attention toward upstream soluble tau intermediates.

## 4. Oxidative Stress: An “Expansionist” Approach

The concept of oxidative stress runs counter to the sensibilities of the practicing neuropathologist because it cannot be directly visualized. An unpaired electron orbiting an atom of oxygen cannot be placed on a glass slide or an electron microscopy grid, and photographed or otherwise imaged. This may explain at least in part its general lesser acceptance as a pathogenic process compared to proteins derived from identifiable, insoluble structural inclusions. As noted above, however, structural inclusions have become something of a nuisance, distracting attention from the putative real culprits—soluble, toxic Aβ or phospho-tau intermediates, themselves invisible as they apparently attack an ultrastructural entity, the synapse, in a molecular process that is further unamenable to direct observation, but rather inferred from electrophysiology or behavioral studies involving the pulling of levers by rats and so forth. In this respect, recent turns toward cell signaling aberrations caused by Aβ oligomers is strategic [3], since cell signaling is more readily measured than an attack on a synapse *in vivo*.

Oxidative damage is thus in the same category as favored hypotheses related to Aβ and tau, in that it is a biochemical phenomenon that cannot be directly observed. It may be argued, however, that the oxidative stress hypothesis offers several conceptual advantages. First, it is well documented that oxidative stress increases with advancing age, in parallel with the strict relationship between age and neurodegenerative disease, along with the general decline in antioxidant defenses [4]. In contrast, Aβ synthesis occurs throughout life and, to date, no mechanism has been offered to explain the absence of amyloidogenesis, over multiple decades, resulting from a putative amyloidogenic cleavage cascade, even in the presence of pathogenic mutations or an extra copy of chromosome 21. Some external factor must exist that has yet to be characterized, for amyloid fibrils, or even amyloid oligomers, to exert a deleterious effect only after years of structurally and functionally normal cellular metabolism. Second, oxidative stress has pleiotropic effects; every category of macromolecule is affected, including membrane lipids, proteins, carbohydrates, and nucleic acids, both DNA and RNA [53,54]. Third, whereas direct damage from unpaired electrons cannot be visualized, oxidative stress results in specific molecular adducts, such as Michael adducts hydroxynonenal, malondialdehyde, advanced glycation adducts pentosidine and pyrraline, heterogeneous adducts such as carboxymethyllysine and nitrotyrosine, and nucleic acid oxidation products such as 8-hydroxyguanosine [55–58]. Such products can be identified and directly measured.

### 4.1. Evidence for oxidative stress

AD increases exponentially throughout life, being non-existent during youth, rare in the middle aged, and common in the elderly [59]. Clearly, age is a risk factor for AD. Like other organ systems, the brain endures a cumulative burden of oxidative damage, which is a universal feature of aging. Moreover, the brain is especially vulnerable to oxidative stress because of its high content of easily peroxidizable fatty acids, high oxygen consumption, and relative paucity of antioxidant enzymes compared with other organs [60–62].

Germline mutations leading to familial early onset AD are also oxidative stress-causing mutations. Increased vulnerability to oxidative stress-induced cell death and/or reduced antioxidant defenses have been demonstrated in: i) cell lines expressing mutant AβPP, PS1, and PS2 [63–66]; ii) transgenic mice expressing mutant AβPP or PS1, and knockout mice expressing mutant human PS1 [67–74]; iii) fibroblasts and lymphoblasts from patients with familial early onset AD with either PS1 or AβPP mutations [75]; iv) cerebral cortex obtained at autopsy from patients with AβPP and PS1 mutations [76,77]; and v) patients with one or both ApoE ɛ4 alleles [78]. *In vitro*, ApoE ɛ4 status shows relative increases in oxidative damage, whereas ApoE ɛ2 status shows relatively decreased oxidative damage [79].

Multiple AD risk factors are associated with an increase in production and propagation of reactive oxygen species, including traumatic brain injury, hypertensive vascular disease, diabetes mellitus, hypercholesterolemia, and hyperhomocysteinemia [80–84]. Environmental and lifestyle related risk factors for AD include aluminum, smoking, high calorie intake, lack of exercise, and lack of intellectual activity [85–88], each of which are associated with oxidative stress and/or reduced antioxidant defenses [89–94]. Decreased risk of AD is further associated with a number of factors including vitamin C and E, estrogen, non-steroidal anti-inflammatory drugs, statins, omega-3 polyunsaturated fatty acids, and red wine [95–100], each of which have antioxidant properties [101–105]. Furthermore, caloric restriction, exercise, and intellectual activity have been shown to promote neuronal survival through enhancement of endogenous antioxidant defenses in experimental animals [106].

### 4.2. Oxidative stress is an early event

The early involvement of oxidative stress in AD is demonstrated by recent studies on cell culture models, transgenic animal models, postmortem brains, and biologic fluids from subjects with AD, MCI, and Down syndrome. Using an *in situ* approach to identify markers of nucleic acid and protein oxidation in human brain, we found that oxidative damage is more pronounced in AD subjects with lesser amounts of Aβ deposition or AD subjects with shorter disease duration [107]. Furthermore, we found that oxidative damage precedes Aβ deposition in brain tissue from Down syndrome, a putative model of AD with predictable neuropathological progression over time [108]. These findings are consistent with the increased nucleic acid oxidation in the brain and in cerebrospinal fluid from AD subjects, in which the shorter the disease duration, the greater the oxidative damage [109–111]. Moreover, individuals with MCI or very mild AD show: i) increased levels of lipid peroxidation and nucleic acid oxidation in postmortem brain tissue [112,113]; ii) increased levels of lipid peroxidation and nucleic acid oxidation in cerebrospinal fluid, plasma, urine, and peripheral leukocytes [114,115]; and iii) decreased levels of plasma antioxidants and total plasma antioxidant capacity [116,117]. Upregulation of heme oxygenase-1, a sensitive marker of oxidative stress, is observed in astroglial cells in postmortem brains of AD and MCI [118].

This human data is supported by experimental studies using cell culture and transgenic animal models of AD. Increased lipid peroxidation and protein oxidation, and decreased Cu/Zn superoxide dismutase activity, precede Aβ deposition or Aβ fibril formation in transgenic mice and *C. elegans* models of AD amyloidosis [69,119–121]. Oxidative stress induces intracellular Aβ accumulation and tau phosphorylation in cell cultures [122–124], and vitamin E reduces Aβ and tau lesions in transgenic animals [125,126]. Further dietary copper stabilizes brain Cu/Zn superoxide dismutase activity and reduces Aβ deposition in AβPP transgenic mice [127]. AβPP mutant mice crossed with manganese SOD heterozygous knockout mice show increased Aβ plaque deposition in the brain [128]. The early involvement of oxidative stress in the pathologic cascade of AD is likely to be closely associated with other key features of AD such as metabolic dysfunction, mitochondrial dysfunction, metal dysregulation, and cell cycle dysregulation [129]. Mitochondrial abnormalities are proving to be a substantial source and recipient of oxidative stress events that likely are responsible for many of the pathological correlates of the cognitive deficiencies including synaptic loss [130]. Aβ and the AβPP molecule both affect mitochondria form and function in cellular and animal models suggesting mitochondria as a key player in the first stages of neuronal dysfunction [131–133]. In fact, all these features are observed as early stage events of AD which are shown in human [134–137] and transgenic animal models [131,138–140]. In short, oxidative stress is not only an upstream event, but also has been shown to play a fundamental, direct role in the pathogenesis of AD by a variety of mechanisms.

### 4.3. Neuropathology equals neuroprotection

As the neuroscience community shifts hypotheses in the direction of soluble intermediates, it is becoming more apparent that the pathology of AD represents effect rather than cause, or response to injury rather than injury *per se*. The presence of significant plaque pathology in the absence of cognitive deficits in humans as noted above, and the absence of neuronal loss in transgenic mice despite prodigious accumulations of Aβ, underscores the secondary nature of Aβ in disease, and a likely fundamental role in antioxidant neuroprotection.

Importantly, whereas high concentrations of Aβ in the micromolar range can lead to oxidative stress in various *in vitro* systems, it is apparent from cell, animal, and human studies that oxidative stress precedes Aβ deposition [69,119,120,126–128]. Moreover, increased density of Aβ plaques is associated with decreased levels of nucleic acid oxidation in postmortem brain tissue from familial and sporadic AD subjects as well as Down syndrome [76,107,108]. These findings further indicate that the process of Aβ plaque formation is linked to protection against oxidative stress. Recently, *in vitro* and *in vivo* studies have demonstrated an antioxidant activity of Aβ. Monomeric Aβ_40_ and Aβ_42_ have been shown to protect cultured neurons from iron and copper-induced oxidative toxicity [141]. In addition, co-injection of iron and Aβ_42_ into rat cerebral cortex is significantly less toxic than infection of iron alone [142]. Moreover, addition of physiological concentrations (low nanomolar range) of Aβ_40_ and Aβ_42_ has been shown to protect lipoproteins from oxidation in cerebrospinal fluid and plasma [143]. These Aβ peptides fail to prevent metal independent oxidation and Aβ_25–35_ lacking the metal binding site located in the N-terminal domain (histidine at positions 6, 13, and 14, and tyrosine at position 10) is less effective at inhibiting oxidation. Therefore, it is likely that the mechanism by which Aβ inhibits oxidation is through binding metal ions, as a form of free radical “sponge” [143]. Pathological lesions, therefore, are a fundamental indicator of neuroprotection and specifically protect brain against heavy metal catalyzed free radical injury.

The antioxidant role of Aβ may also apply to tau. Cellular, animal, and human studies suggest that, like Aβ deposition, oxidative stress precedes neurofibrillary tangle formation [108,124,125]. Oxidative stress activates several kinases, including glycogen synthase kinase-3 and mitogen-activated protein kinases, which are activated in AD and capable of phosphorylating tau. Once phosphorylated, tau becomes vulnerable to oxidative modification, formation of intra- and intermolecular crosslinks, and aggregation into filaments (reviewed in [144]). Neurofibrillary tangle formation therefore is accelerated by oxidative stress, which is consistent with evidence of induction of the antioxidant enzyme, heme oxygenase-1 [145]. Furthermore, in postmortem AD tissue, nucleic acid oxidation is decreased in neurofibrillary tangle-containing neurons compared to vulnerable neurons without neurofibrillary tangles [107]. Although the mechanism by which neurofibrillary tangle formation potentially reduces oxidative stress is unknown, redox active iron accumulation within neurofibrillary tangles has been demonstrated [146], suggesting, in analogy to Aβ plaques, that neurofibrillary tangles are a sink for heavy metal derived free radicals.

In short, recent data with respect to Aβ and tau suggest insoluble pathological lesions, rather than being toxic as previously thought, are non-toxic responses to the underlying disease process that, in essence, are indicators of the hosts attempt at antioxidant neuroprotection.

## 5. Conclusions

Current theories pertaining to the molecular pathogenesis of AD encompass copious and sophisticated data, but nevertheless have their origin, or foundation, in hallmark pathological lesions described more than a century ago. Once considered manifestations of neurotoxicity, these same lesions are now considered insoluble epiphenomena that only distract from upstream pathophysiological events involving soluble, toxic intermediates and the synapse, events ironically that cannot be directly observed. For better or worse, the lesions of origin, in essence the microscopic neuropathology, are now discarded in the pellet of the ultracentrifuge tube, during the course of study of the molecular pathogenesis of AD. Based on considerable evidence, however, we favor the few that pathological lesions can be tied to molecular pathogenesis on the basis of disease expression, or a host response, that is fundamentally adaptive over a long period of time, to combat the adverse pleiotropic effects of oxidative damage. So-called toxic intermediates may well be an extension of this productive host response and should be considered for therapeutic targeting only with considerable care. The time for a paradigm shift that targets the more expansionist approach of oxidative stress, rather than reductionist approaches centered on singular small molecules, may be warranted, and in fact overdue.
